# Community-level group sports participation and all-cause, cardiovascular disease, and cancer mortality: a 7-year longitudinal study

**DOI:** 10.1186/s12966-024-01592-9

**Published:** 2024-04-24

**Authors:** Taishi Tsuji, Eisaku Okada, Masashige Saito, Satoru Kanamori, Yasuhiro Miyaguni, Masamichi Hanazato, Katsunori Kondo, Toshiyuki Ojima

**Affiliations:** 1 Institute of Health and Sport Sciences, University of Tsukuba, 3-29-1 Otsuka, 112-0012 Bunkyo, Tokyo Japan; 2 Center for Preventive Medical Sciences, Chiba University, 1-33 Yayoi-cho, Inage Ward, 263-8522 Chiba, Japan; 3 Faculty of Social Policy & Administration, Hosei University, 4342, Aihara, 194-0298 Machida, Tokyo Japan; 4 Department of Social Welfare, Nihon Fukushi University, Okuda, Mihama-cho, 470-3295 Chita-gun, Aichi Japan; 5 Center for Well-being and Society, Nihon Fukushi University, 5-22-35 Chiyoda, Naka Ward, 460-0012 Nagoya, Aichi Japan; 6 Teikyo University Graduate School of Public Health, 2-11-1 Kaga, 173-8605 Itabashi, Tokyo Japan; 7 Department of Preventive Medicine and Public Health, Tokyo Medical University, 6-1-1 Shinjuku, 160-8402 Shinjuku, Tokyo Japan; 8 Center for Gerontology and Social Science, Research Institute, National Center for Geriatrics and Gerontology, 7-430 Morioka-cho, 474-8511 Obu, Aichi Japan; 9 Department of Community Health and Preventive Medicine, Hamamatsu University School of Medicine, 1-20-1 Handayama, Chuo Ward, 431-3192 Hamamatsu, Shizuoka Japan

**Keywords:** Social participation, Social capital, Contextual effect, Japan, Older adults

## Abstract

**Background:**

Community-level group sports participation is a structural aspect of social capital that can potentially impact individual health in a contextual manner. This study aimed to investigate contextual relationship between the community-level prevalence of group sports participation and the risk of all-cause, cardiovascular disease (CVD), and cancer mortality in older adults.

**Methods:**

In this 7-year longitudinal cohort study, data from the Japan Gerontological Evaluation Study, a nationwide survey encompassing 43,088 functionally independent older adults residing in 311 communities, were used. Cause of death data were derived from the Japanese governmental agency, The Ministry of Health, Labour and Welfare, for secondary use. “Participation” was defined as engaging in group sports for one or more days per month. To analyze the data, a two-level survival analysis was employed, and hazard ratios (HRs) and 95% confidence intervals (CIs) were calculated.

**Results:**

Among the participants, 5,711 (13.3%) deaths were identified, with 1,311 related to CVD and 2,349 to cancer. The average group sports participation rate was 28.3% (range, 10.0–52.7%). After adjusting for individual-level group sports participation and potential confounders, a higher community-level group sports participation rate was found to be significantly associated with a lower risk of both all-cause mortality (HR: 0.89, 95% CI: 0.83–0.95) and cancer mortality (HR: 0.89, 95% CI: 0.81–0.98) for every 10% point increase in the participation rate. For CVD mortality, the association became less significant in the model adjusted for all covariates (HR: 0.94, 95% CI: 0.82–1.09).

**Conclusions:**

Our findings support the existence of a preventive relationship between community-level group sports participation and the occurrence of all-cause and cancer mortality among older individuals. Promoting group sports within communities holds promise as an effective population-based strategy for extending life expectancy, regardless of individual participation in these groups.

**Supplementary Information:**

The online version contains supplementary material available at 10.1186/s12966-024-01592-9.

## Background

Engaging in sports and exercise as part of a group has several advantages for older adults, including a reduced risk of functional disability [1], depressive symptoms [2], and falls [3], compared to participating in individual sports and exercise without group involvement. The health outcomes achieved through group participation in sports as opposed to individual exercise may be attributed to several factors. These factors include the benefits of physical activity, such as promoting good adherence and a longer duration of exercise. Furthermore, psychological factors such as increased enjoyment, enhanced self-esteem, and decreased stress and social factors, including receiving social support, social capital, and social influence, also play a significant role [4]. 

Community-level group sports participation has been shown to significantly reduce depressive symptoms [5] and cognitive impairment [6] in older adults. Therefore, older adults residing in communities with higher group sport participation rates, regardless of participation at the individual level, are less likely to exhibit depressive symptoms and cognitive impairment compared to those residing in communities with lower participation rates. These findings may be attributed to the contextual effects facilitated by the presence of social capital within the community [7]. Previous studies [7–9] have provided a comprehensive theoretical overview of social capital and described it as “features of social organizations, such as networks, norms, and trust, that facilitate action and cooperation for mutual benefit” [10] or “resources that are accessed by individuals as a result of their membership of a network or a group.” [7] Communities where group sports thrive typically exhibit higher community-level social capital, which, in turn, can facilitate adoption of healthy behaviors among individuals who do not personally participate in the group sports through social contagion, informal social control, and collective efficacy in the community [7]. The empirical evidence from the following studies supports the existence of this effect. Older adults, including those who do not participate in group sports, residing in a community with higher group sport participation rates among older adults are less likely to be homebound and exhibit higher transtheoretical model stages of change for exercise compared to those residing in communities with lower participation rates [11]. 

As previously mentioned, the beneficial effects of community-level group sports participation on health behaviors and status among older adults have been established. However, it is still unclear whether this participation can actually reduce the risk of mortality. A systematic review was conducted to investigate the relationship between social capital and mortality, including all-cause as well as cause-specific (i.e., cardiovascular disease [CVD] and cancer) mortality [12]. This review revealed a lack of studies specifically focusing on community-level social participation, which pertains to the structural aspects of social capital. Accordingly, we conducted this study to investigate the potential association between community-level group sports participation and the risk of all-cause, CVD, and cancer mortality among older adults. We hypothesized that older adults living in communities with higher group sports participation rates have a lower risk of mortality due to each cause than those living in communities with lower group sports participation rates. If this hypothesis is supported, the promotion of community group sports could be used as an effective population-based strategy for extending life expectancy, regardless of their participation in group sports.

## Methods

### Study design and participants

**Fig. 1:**
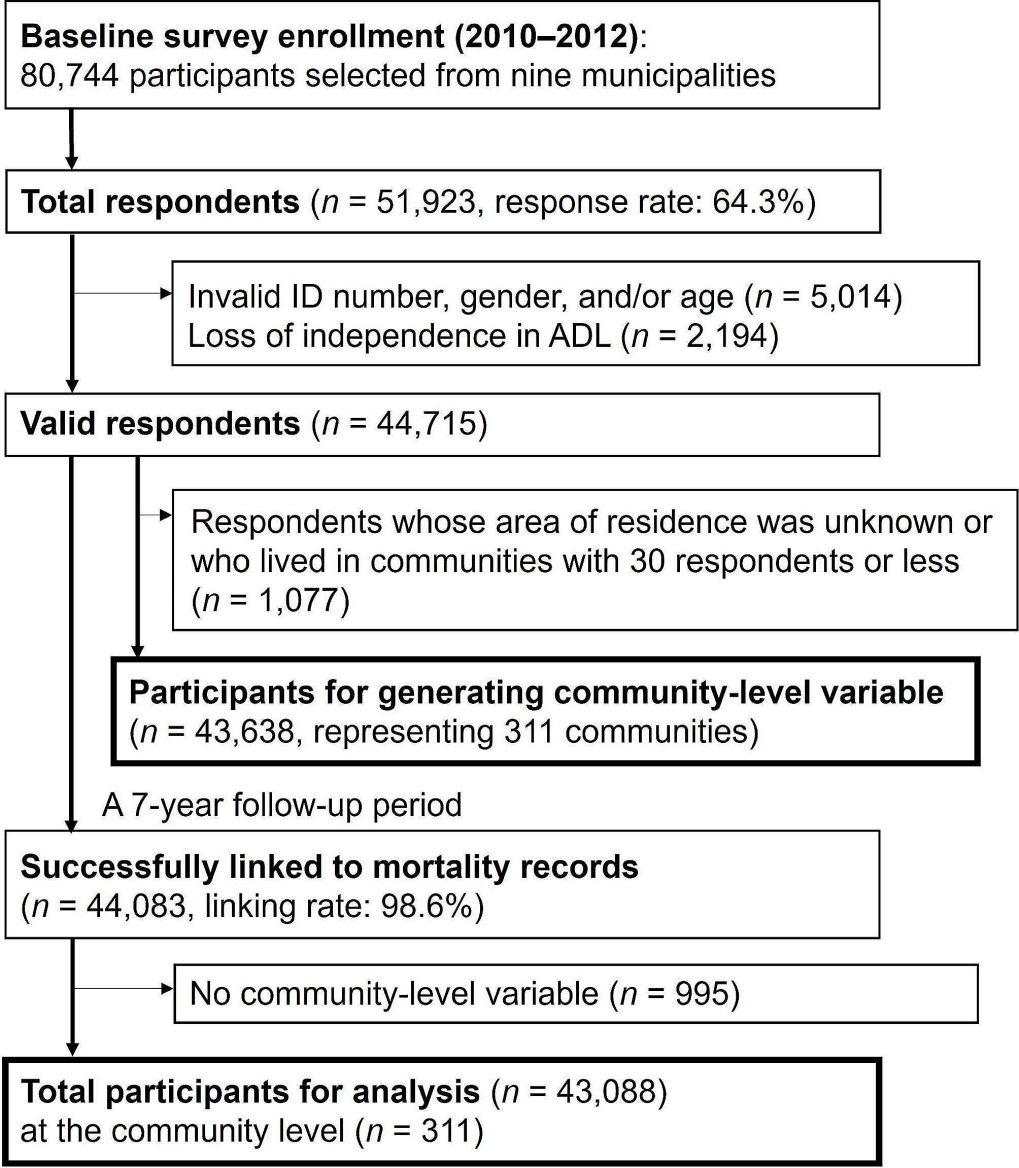
Participants’ flow in the cohort study

This study utilized longitudinal cohort data from the Japan Gerontological Evaluation Study (JAGES) [13, 14]. JAGES is an ongoing investigation that focuses on examining social, behavioral, and environmental factors associated with functional decline or cognitive impairment in individuals aged 65 or older, which can lead to a loss of independence. The participant flow for this study is depicted in Fig. 1. The baseline survey was conducted between August 2010 and January 2012. During this time frame, self-reported questionnaires were mailed to a total of 80,744 community-dwelling individuals aged 65 or older who were both physically and cognitively independent. These participants were selected from nine municipalities in six prefectures across Japan. A random sample was drawn from the official residence registers in four municipalities, while the remaining five smaller municipalities conducted a complete census of their older residents. Out of the 51,923 respondents (response rate: 64.3%), valid information on ID, gender, age, and ability to perform activities of daily living was collected from 44,715 respondents (valid response rate: 55.4%). To obtain the community-level group sports participation variable, 1,077 respondents with an unknown area of residence or those residing in community areas with 30 or fewer respondents were excluded. This was done to ensure the accuracy of the data by excluding small sample sizes, resulting in 43,638 respondents nested in 311 areas defined primarily by school districts. This provided a valid sample for creating the community-level variable. During the construction of cohort data to track individuals, 44,083 of the 44,715 valid respondents (98.6%) were successfully linked to mortality records over a 7-year follow-up period, which ended on December 31, 2017. For the present multilevel survival analyses, a sample of 43,088 participants (20,186 men and 22,902 women) was analyzed after excluding respondents who lacked information on the community-level variable.

The current study was conducted in accordance with the principles of the Helsinki Declaration and approved by the Ethics Committee, Chiba University, Japan (approval number: 2493), and the National Center for Geriatrics and Gerontology, Japan (approval number: 992-3). All participants were informed about the voluntary nature of their participation and indicated their consent by completing and returning the questionnaire *via* mail.

### Mortality

Information on the date of death was retrieved from the public long-term care insurance system database administered by the municipal governments. The data on the cause of death were then derived from the secondary use of information maintained by the Ministry of Health, Labour and Welfare, a Japanese governmental agency. In Japan, the cause of death is determined based on the death certificate completed by physicians and then reported to the government. Using the information provided about the participants’ area of residence, date of birth, and date of death, the mortality data were linked to the baseline self-reported questionnaire data. The outcomes, including all-cause, CVD-related (I00-I99), and cancer-related (C00-D48) mortality, were assessed according to the 10th revision of the International Classification of Diseases (ICD-10) [15]. 

### Community- and individual-level independent variables

Participants were evaluated based on their frequency of group sports participation, with response options ranging from four days per week or more to 2–3 days per week, one day per week, 1–3 days per month, a few times per year, or zero. Consistent with previous studies [6, 16], “participation” was defined as engaging in group sports activity at least one day per month. To create a community-level independent variable, individual-level group sports participation data were aggregated by community area. In this regard, we calculated the group sports participation rates by dividing the number of group sports participants by the total number of participants included in the analysis. Previous research has suggested a stronger correlation between the proportion of older individuals reporting poor self-rated health or experiencing depressive symptoms and community-level group sports participation occurring frequently at least one day per month (*r* = − 0.233 and − 0.355, respectively) compared to participation occurring frequently at least one day per week (*r* = − 0.210 and − 0.314, respectively) [16]. 

### Covariates

Based on previous studies examining the association between community-level group sports participation and health behaviors and status among older adults [5, 6, 11], several covariates were collected. Information on gender and age was collected as basic demographic characteristics, with age groups categorized as 65–69, 70–74, 75–79, 80–84, or ≥ 85 years. In terms of community-level covariates, population density per km^2^ of inhabitable area was calculated for each community area using national statistics data and categorized into quartile categories (≥ 10,350, 8,190–10,349, 5,680–8,189, or < 5,680 persons per km^2^). In terms of individual-level covariates, marital status (married or unmarried), living alone (no or yes), occupational status (employed, retired, or never employed), years of education (≥ 13, 10–12, or < 10 years), alcohol drinking status (none, past, or current), and smoking status (none, past, or current) were collected. Annual equivalent income was calculated by dividing household income by the square root of the number of household members and categorized into three groups: $40,000 or more; $20,000–$39,999; or less than $20,000 per year (1 Dollar = 100 Yen). Body mass index (BMI) was calculated using self-reported height and weight (kg/m^2^) and categorized as underweight (< 18.5), normal weight (18.5–24.9), or overweight/obese (≥ 25.0). BMI values ≥ 25 were grouped into one category as only a small proportion of older adults in Japan and only 1.9% of the current study sample were considered to be obese (i.e., BMI ≥ 30). Disease status was assessed by yes or no responses and included hypertension, stroke, heart disease, diabetes mellitus, hyperlipidemia, musculoskeletal disorders, and cancer. Self-rated health was assessed on a 4-point Likert scale (very good, good, somewhat poor, or poor). Depressive symptoms were assessed using the short version of the 15-item Geriatric Depression Scale [17, 18] and categorized into three groups: none (0–4 points), mild (5–9 points), or moderate to severe (10–15 points). Instrumental activities of daily living were assessed using the Tokyo Metropolitan Institute of Gerontology Index of Competence [19] and classified as good (5 points) or poor (≤ 4 points). Daily walking time was classified into four groups, as follows: <30, 30–59, 60–89, or ≥ 90 min. For the subgroup analysis, the study sample was further classified into two categories based on whether they met the recommended number of steps for older adults (i.e., 6,000–7,000 steps/day, as recommended by the national health promotion program in Japan), as follows: (a) did not meet criteria—walking duration per day < 60 min; and (b) met the criteria—walking duration per day > 60 min [20, 21]. 

### Statistical analyses

Since there was no statistically significant interaction between community-level group sports participation and gender for each mortality outcome, the analysis was conducted on the entire participant population without stratification by gender. The multilevel analysis framework assumes that individual outcomes are influenced in part by the community areas in which individuals reside. In this study, multilevel survival analysis was used to calculate hazard ratios (HRs) and 95% confidence intervals (CIs) for mortality during the follow-up period. The HR of community-level group sports participation was estimated based on a 10-percentage point change in the aggregated percentages of group sports participation. Three multivariate models were employed for the analyses. The crude model included both community-level and individual-level group sports participation, as well as cross-level interaction terms. Model 1 consecutively incorporated age group and gender, while Model 2 sequentially included all covariates except daily walking time. To address potential bias resulting from missing values, multiple imputations were performed under the assumption of missingness at random (i.e., missingness is related to other variables measured in the same survey for each participant). Incomplete variables were imputed using a multivariate normal imputation method. A total of 20 imputed datasets were created, encompassing all variables used in the present analyses. The estimated parameters were combined using Rubin’s combination methods [22, 23]. To ensure the robustness of the results, a sensitivity analysis was conducted by excluding individuals with a history of stroke, heart disease, or cancer at baseline. Additionally, subgroup analyses stratified by daily walking time (i.e., < 60 or ≥ 60 min) were performed. Supplementary analyses were conducted to explore the dose–response relationship between the frequency of individual-level participation in group sports (ref. zero vs. a few times per year, 1–3 days per month, and 1 day per week or more) and each mortality outcome. All statistical analyses were performed using STATA/MP 17.0 (Stata Corp., College Station, TX, USA).

## Results

Table 1 summarizes the descriptive data for individual- and community-level variables as well as cumulative mortality. The present study included 43,088 participants, contributing a total of 272,347 person-years. The maximum follow-up duration was 2,709 days, and the mean follow-up duration was 2,309 days. A total of 5,711 deaths (13.3%) were identified, with 1,311 related to CVD and 2,349 related to cancer. The mortality rates per 1,000 persons per year were 21.0 for all-cause mortality, 4.8 for CVD mortality, and 8.6 for cancer mortality. After applying multiple imputations, the estimated group sports participation rate was calculated for each community area. The average participation rate was 28.3%, with a standard deviation of 7.0% and a range of 10.0–52.7%.

**Table 1:** Descriptive statistics for individual- and community-level variables as well as cumulative mortality

Individual-level variables	Total *n*	%	
**Total**	43,088		
**All-cause mortality**	5,711	13.3%	
**Cardiovascular mortality**	1,311	3.0%	
**Cancer mortality**	2,349	5.5%	
**Participation in group sports**			
Zero	24,277	56.3%	
A few times/year	1,505	3.5%	
1–3 days/month	1,673	3.9%	
1 day/week	2,601	6.0%	
2–3 days/week	3,248	7.5%	
≥ 4 days/week	860	2.0%	
Missing	8,924	20.7%	
**Participation in group sports (frequently at least one day per month)**			
No	25,782	59.8%	
Yes	8,382	19.5%	
Missing	8,924	20.7%	
**Gender**			
Male	20,186	46.8%	
Female	22,902	53.2%	
**Age (years)**			
65–69	12,779	29.7%	
70–74	12,861	29.8%	
75–79	9,616	22.3%	
80–84	5,402	12.5%	
≥ 85	2,430	5.6%	
**Marital status**			
Married	30,637	71.1%	
Unmarried	11,752	27.3%	
Missing	699	1.6%	
**Living alone**			
No	36,823	85.5%	
Yes	5,581	13.0%	
Missing	684	1.6%	
**Education (years)**			
≥ 13	7,824	18.2%	
10–12	15,032	34.9%	
< 10	19,212	44.6%	
Missing	1,020	2.4%	
**Annual equivalent income (Yen)**			
≥ 4,000,000	3,975	9.2%	
2,000,000–3,999,999	14,214	33.0%	
< 2,000,000	19,782	45.9%	
Missing	5,117	11.9%	
**Occupational status**			
Employed	8,820	20.5%	
Retired	24,330	56.5%	
Never employed	4,765	11.1%	
Missing	5,173	12.0%	
**Drinking status**			
None	14,737	34.2%	
Past	1,415	3.3%	
Current	24,468	56.8%	
Missing	2,468	5.7%	
**Smoking status**			
None	23,023	53.4%	
Past	11,540	26.8%	
Current	4,421	10.3%	
Missing	4,104	9.5%	
**Body mass index**			
< 18.5	2,971	6.9%	
18.5–24.9	29,306	68.0%	
≥ 25	9,140	21.2%	
Missing	1,671	3.9%	
**Disease status in treatment**			
Hypertension	17,126	39.7%	
Stroke	516	1.2%	
Heart disease	5,280	12.3%	
Diabetes mellitus	5,438	12.6%	
Hyperlipidemia	4,261	9.9%	
Musculoskeletal disorders	6,891	16.0%	
Cancer	1,913	4.4%	
Missing	842	2.0%	
**Self-rated health**			
Excellent	5,144	11.9%	
Good	29,598	68.7%	
Poor or very poor	7,886	18.3%	
Missing	460	1.1%	
**Depressive symptoms**			
No	30,349	70.4%	
Yes	11,955	27.7%	
Missing	784	1.8%	
**Instrumental activities of daily living**			
Good	32,720	75.9%	
Poor	7,611	17.7%	
Missing	2,757	6.4%	
**Daily walking time (minutes)**			
< 60	27,783	64.5%	
≥ 60	12,732	29.5%	
Missing	2,573	6.0%	
**Community-level variables**	n		
**Total**	311		
Estimated group sports participation rate*			
Mean (SD)		28.3%	(7.0%)
(Min–max)		(10.0–52.7%)	
**Population density (persons per km** ^**2**^ **of inhabitable area)**			
Highest quartile (≥ 10,350)	78		
2nd quartile (8,190–10,349)	78		
3rd quartile (5,680–8,189)	78		
Lowest quartile (< 5,680)	77		

SD, standard deviation

*Calculated after applying multiple imputations

Table 2 summarizes the results from the multilevel survival analyses. In the crude model and the models adjusted by gender and age (Model 1), a higher community-level group sports participation rate was associated with a reduced risk of all mortality outcomes. In the fully adjusted model (Model 2), a higher participation rate was significantly associated with a lower risk of all-cause mortality (HR: 0.89, 95% CI: 0.83–0.95) and cancer mortality (HR: 0.89, 95% CI: 0.81–0.98) for every 10% point increase in the participation rate. Individual-level group sports participation was also found to lower the risk of all mortality outcomes across all models. However, no significant cross-level interactions were identified. Supplementary Table 1 presents the results of the sensitivity analysis, excluding participants with a history of stroke, heart disease, or cancer at baseline. Supplementary Table 2 shows the results of the subgroup analyses stratified by daily walking time (i.e., < 60 or ≥ 60 min). These results were comparable to those observed in the main analysis.

**Table 2:** Results of multilevel survival analyses for all-cause, cardiovascular, and cancer mortality

	Crude model				Model 1				Model 2			
	HR	95% CI		*p*	HR	95% CI		*p*	HR	95% CI		*p*
**All-cause mortality**												
Group sports participation rate*	0.83	0.78	0.88	< 0.001	0.84	0.79	0.90	< 0.001	0.89	0.83	0.95	< 0.001
Participation in group sports†	0.60	0.54	0.65	< 0.001	0.66	0.61	0.72	< 0.001	0.77	0.70	0.84	< 0.001
Cross-level interaction	0.91	0.78	1.06	0.231	0.91	0.78	1.06	0.223	0.92	0.79	1.07	0.267
**Cardiovascular mortality**												
Group sports participation rate*	0.84	0.75	0.95	0.004	0.87	0.77	0.98	0.025	0.94	0.82	1.09	0.425
Participation in group sports†	0.58	0.48	0.69	< 0.001	0.65	0.54	0.78	< 0.001	0.77	0.64	0.93	0.006
Cross-level interaction	0.92	0.67	1.26	0.584	0.91	0.67	1.24	0.565	0.91	0.66	1.24	0.540
**Cancer mortality**												
Group sports participation rate*	0.86	0.79	0.94	0.001	0.86	0.79	0.94	0.001	0.89	0.81	0.98	0.015
Participation in group sports†	0.66	0.58	0.75	< 0.001	0.71	0.63	0.81	< 0.001	0.79	0.70	0.91	0.001
Cross-level interaction	0.90	0.71	1.13	0.371	0.90	0.72	1.14	0.382	0.91	0.72	1.16	0.454

Data comprising 43,088 participants from 311 community areas

HR, hazard ratio; CI, confidence interval

*HR for a 10% point increment in group sports participation rate in a community area

†HR for participating in group sports frequently, at least one day per month, vs. participating less frequently than one day per month

Model 1: crude model + age and gender

Model 2: Model 1 + all covariates except daily walking time

Supplementary Table 3 presents the results of supplementary analyses examining the dose–response relationship between the frequency of individual-level participation in group sports and each mortality outcome. Across all causes of mortality, increased frequency of individual-level participation in group sports was associated with a lower mortality risk.

## Discussion

To the best of our knowledge, this is the first study to investigate the contextual relationship between the community-level prevalence of group sports participation and mortality among older adults. After adjusting for individual-level group sports participation and potential confounders, a 10-percentage point increase in community-level group sports participation rate was found to be associated with an 11% decrease in the risks of all-cause and cancer-related mortality. The results were found to be robust regardless of the daily walking time. These findings suggest that promoting group sports within the community could be an effective strategy for extending life expectancy, benefiting both those who actively participate in the groups and those who do not.

A study conducted in England found that higher levels of social activity, including participation in sports clubs, at the community level were associated with a reduced risk of all-cause mortality among adults residing in the community [24]. Similar findings were reported in our study of older population in Japan. We hypothesize that the contextual effect of group-level social capital on individual-level health outcomes can be explained through three pathways: social contagion, informal social control, and collective efficacy. Social contagion suggests that behaviors spread more rapidly within tightly knit social networks [7], promoting healthy lifestyle changes such as smoking cessation [25]. Informal social control refers to the ability of individuals in a community to maintain social order, for instance, by intervening when they witness deviant behavior [7]. In the context of this study, group sports participants may encourage older individuals, whether engaged in individual or group-based activities, to engage in more physical activity. Collective efficacy, similar to the concept of self-efficacy at the group level, refers to the collective’s ability to mobilize and take collective action [7, 26]. Communities with numerous group sports and participants may develop facilities, industries, systems, or regulations that promote health and align with the community’s opinions and actions. The widespread participation in group sports at the group level may have positive spillover effects [5]. Based on the transtheoretical model, older adults residing in communities with high group sports participation rates, even if they do not participate themselves, were less likely to be homebound and had higher stages of change for exercise than those residing in communities with low participation rates [11]. 

In terms of causes of mortality, divergent trends were observed between CVD and cancer. In the case of CVD mortality, the association became less significant in the model adjusted for covariates, whereas it remained significant in the case of cancer-related mortality. This suggests that communities with higher group sports participation rates may have a lower risk of CVD mortality due to “compositional effects.” These effects are associated with residing in communities where a greater proportion of older adults possess favorable individual factors, which were also adjusted for in Model 2 (e.g., socioeconomic status, health behaviors, and health status). In contrast, for cancer-related mortality, the contextual effects identified for all-cause mortality may persist irrespective of these individual factors. A study conducted in Sweden [27], which included all individuals aged 65 and older, demonstrated that low community-level social capital, as measured by voting participation rates, was linked to an elevated risk of cancer, coronary heart disease, and stroke. However, the Swedish report only adjusted for socioeconomic factors such as income, educational level, and marital status, rather than individual health behaviors and status, as was done in the present study. Further research is warranted to elucidate the mechanisms by which the level of social and civic participation at the community level influences the risk of mortality from specific causes.

The present study has several strengths. First, the study included a large sample of older adults from nine municipalities across a diverse range of urban settings nationwide. Second, extensive longitudinal data were obtained from national certification records, resulting in a high follow-up rate over a 7-year period. However, certain limitations should be acknowledged. First, due to the limited number of events, the analysis did not include a detailed breakdown of the causes of mortality for CVD and cancer. It is worth noting that a report of Swedish adults found a consistent negative association between community-level social capital, as measured by voting participation rates, and mortality from lung [28] and colorectal cancers [29] in men and women, as well as prostate cancer [30] in men. Second, the results may be susceptible to selection bias due to the relatively low valid response rate (55.4%). According to a previous study, both the response rate and the percentage of group sports participants were significantly lower among older individuals than among younger individuals [31]. As a result, the participants in this study may have had a relatively lower risk of mortality, potentially underestimating the relationship. Third, the influence of unmeasured confounders, particularly community-level variables that were not adequately accounted for, cannot be ignored. Future investigations into available open data using geographic information systems are required to examine the impact of the physical environment more comprehensively.

## Conclusions

After adjusting for individual-level group sports participation and other covariates, older adults residing in communities with higher group sports participation rates have a lower risk of all-cause and cancer-related mortality than those residing in communities with lower group sports participation rates. These findings indicate a contextually preventive association between community-level group sports participation and all-cause and cancer-related mortality among older individuals. The promotion of group sports within a community may be an effective population-based strategy for extending life expectancy, benefiting both those who actively participate in group sports and those who do not.

### Electronic supplementary material

Below is the link to the electronic supplementary material.

Supplementary Material 1

Supplementary Material 2

Supplementary Material 3

Supplementary Material 4

## Data Availability

The data generated and/or analysed during the current study are not publicly available due to it sensitive nature and source of origin being the JAGES but are available from the JAGES on reasonable request. Requests for JAGES data can be sent to dataadmin.ml@jages.net.

## Abbreviations

BMI: Body mass index
CI: Confidence intervals
CVD: Cardiovascular disease
HR: Hazard ratios
JAGES: Japan Gerontological Evaluation Study
